# Eosinophils in the Lung – Modulating Apoptosis and Efferocytosis in Airway Inflammation

**DOI:** 10.3389/fimmu.2014.00302

**Published:** 2014-07-01

**Authors:** Jennifer M. Felton, Christopher D. Lucas, Adriano G. Rossi, Ian Dransfield

**Affiliations:** ^1^MRC Centre for Inflammation Research, Queen’s Medical Research Institute, The University of Edinburgh, Edinburgh, UK

**Keywords:** eosinophil, lung, inflammation, apoptosis, phagocytosis, allergy, airway, resolution

## Abstract

Due to the key role of the lung in efficient transfer of oxygen in exchange for carbon dioxide, a controlled inflammatory response is essential for restoration of tissue homeostasis following airway exposure to bacterial pathogens or environmental toxins. Unregulated or prolonged inflammatory responses in the lungs can lead to tissue damage, disrupting normal tissue architecture, and consequently compromising efficient gaseous exchange. Failure to resolve inflammation underlies the development and/or progression of a number of inflammatory lung diseases including asthma. Eosinophils, granulocytic cells of the innate immune system, are primarily involved in defense against parasitic infections. However, the propagation of the allergic inflammatory response in chronic asthma is thought to involve excessive recruitment and impaired apoptosis of eosinophils together with defective phagocytic clearance of apoptotic cells (efferocytosis). In terms of therapeutic approaches for the treatment of asthma, the widespread use of glucocorticoids is associated with a number of adverse health consequences after long-term use, while some patients suffer from steroid-resistant disease. A new approach for therapeutic intervention would be to promote the resolution of inflammation via modulation of eosinophil apoptosis and the phagocytic clearance of apoptotic cells. This review focuses on the mechanisms underpinning eosinophil-mediated lung damage, currently available treatments and therapeutic targets that might in future be harnessed to facilitate inflammation resolution by the manipulation of cell survival and clearance pathways.

## Introduction

In response to tissue injury or the presence of micro-organisms, initiation of host protective mechanisms associated with the acute inflammatory response can also cause damage to the surrounding tissue. The release of proteases, glycosidases, and reactive oxygen/nitrogen species can be particularly destructive in the lung, where disruption of the normal tissue architecture compromises efficient gaseous exchange. A corollary of this close relationship between inflammation and tissue injury is that successful resolution of inflammation is crucial to optimal restoration and maintenance of lung function.

The detection of pathogen-associated molecular patterns (PAMPs) or danger-associated molecular patterns (DAMPs) via their cognate receptors leads to the production of pro-inflammatory mediators including tumor necrosis factor-alpha (TNF-α), interleukin (IL)-1, and IL-6. Well characterized chemoattractants such as complement fragments (e.g., C3a and C5a), lipids [e.g., leukotriene B_4_ (LTB_4_) and platelet-activating factor (PAF)], and chemokines [e.g., IL-8 (CXCL8), MCP-1 (CCL2), and eotaxin (CCL11)] act to recruit and/or activate inflammatory cells. Together these mediators rapidly perpetuate inflammation via the activation of vascular endothelial cells, increased vascular permeability, and edema, concurrent with the recruitment of granulocytes at the site of injury. In this review, we will discuss the mechanisms controlling acute lung inflammation, pathological conditions where the regulation of inflammation has gone awry and discuss the current and future treatments that could promote the successful resolution of inflammation.

### Polymorphonuclear granulocytes: Critical effectors of the innate immune response

Neutrophils and eosinophils are key immune cells in the host defense against invading bacteria and parasites. Excessive recruitment, uncontrolled activation, and defective removal of these cells play a prominent role in the initiation and propagation of a number of chronic inflammatory conditions (1). Apoptosis, a major form of programed cell death, is a fundamental process regulating the tissue longevity of inflammatory cells. Apoptosis provides an efficient non-inflammatory mechanism for the removal of potentially damaging cells and cellular content from the inflamed site by resident or recruited monocyte/macrophage populations (2) or by “non-professional” phagocytes such as epithelial cells (3). The observation of failed apoptotic cell clearance seen in a number of chronic inflammatory diseases, including asthma, bronchiectasis, and chronic obstructive pulmonary disease (COPD) provides strong evidence that granulocyte apoptosis and non-inflammatory clearance has a key role in the resolution of inflammation.

Neutrophils are continuously generated from pluripotent stem cells in the bone marrow and are released into the circulation in large numbers [up to 2 × 10^11^ cells/day (4)]. Once appropriately triggered, circulating neutrophils or those mobilized from the large marginated pools in the lungs, liver, spleen, and bone marrow (5, 6), can be rapidly recruited to the inflammatory site and engage a number of effector mechanisms to destroy invading pathogenic organisms. This distinctive machinery includes a combination of reactive oxygen species (ROS) generation, the release of a cocktail of cytotoxic and proteolytic molecules, phagocytosis, and NETosis (the formation of extracellular chromatin traps) to destroy invading pathogenic organisms. Recruited neutrophils can undergo apoptosis which is associated with the “disabling” of secretion of their potentially harmful granule content thereby preventing damage to the surrounding tissues (7). The removal of these apoptotic cells by recruited macrophages or other local phagocytes, including airway epithelial cells (3), is believed to facilitate the resolution of inflammation. In addition to apoptotic cell death, a number of other forms of neutrophil cell death have been documented, including necrosis, NETosis, autophagocytic cell death, necroptosis, oncosis, and pyroptosis [reviewed in Ref. (8, 9)]. Although the impact of these alternative forms of cell death on the resolution of inflammatory responses is less clear, several are believed to be predominantly pro-inflammatory. As well as local cell death recent studies have provided evidence that recruited granulocytes can also undergo reverse migration away from the site of inflammation (10–12), although the consequences of this on inflammatory processes requires further investigation.

Eosinophils are also derived from granulocytic precursor populations in the bone marrow and are readily recruited from residence within hematopoietic and lymphatic organs such as the lymph nodes, thymus, spleen, and bone marrow (13) via the vasculature to the site of injury in response to parasitic or allergic inflammation (14). Historically, these cells were considered to play little role in immunoregulation, however, several lines of investigation have now shown eosinophils to be multifunctional granulocytes involved in the initiation and propagation of numerous inflammatory responses, including modulation of the adaptive immune response (14). Once at the site of injury, eosinophil degranulation contributes to both the removal of the inflammatory stimuli and also the propagation of inflammation. Eosinophil-derived granules contain a wide range of proteins including, major basic protein, eosinophil cationic protein, eosinophil peroxidase, and eosinophil-derived neurotoxin, which are known to be cytotoxic to airway epithelial cells (15, 16). Eosinophils have also been shown to undergo “traditional” extracellular trap formation (ETosis) termed EETosis (17) as well as facilitating the extracellular release of mitochondrial chromatin in a ROS-dependent manner (13). Released mitochondrial DNA and eosinophil-derived granule proteins combine to form structures, which are capable of both binding and killing invading organisms *in vitro* and *in vivo* (13), indicating that eosinophils may play a previously unrecognized role in antimicrobial defense. The fate of tissue eosinophils includes apoptosis (18) and subsequent clearance by phagocytes, although alternative fates have also been reported.

### Apoptotic pathways

There are two major pathways of apoptosis. The *intrinsic* pathway is characterized by a conformational change in pro-apoptotic Bcl-2 protein family members, resulting in outer mitochondrial membrane pore formation. The subsequent release of cytochrome *c* leads to formation of a complex with apoptotic protease-activating factor-1 (APAF-1), which then activates the downstream caspases that facilitate apoptosis. In contrast, the *extrinsic* pathway is triggered by cell surface death receptor trimerization resulting in the activation of Fas-associated protein with death domain (FADD) and TNF-receptor type 1-associated death domain protein (TRADD), which is responsible for the autocatalytic activation of initiator and effector caspases leading to the synchronized molecular alterations and morphological changes associated with apoptosis. Thus, the result of these two divergent pathways is the activation of intracellular caspases (a family of cysteine–aspartic proteases), which represents a hallmark event in apoptosis [reviewed in Ref. (8, 19, 20)].

## Airway Inflammation

### Normal lung structure

The lung is made of up three distinctly different anatomical areas, the proximal cartilaginous airways, distal bronchioles, and alveoli (21). The trachea and main bronchi form the proximal cartilaginous airways and are responsible for the conduction of inhaled air, during which the proximal pseudostratified epithelium provides defense against invading pathogens and environmental toxins. In contrast, the epithelium of the distal airways becomes more columnar and is populated by a large number of ciliated epithelial cells and mucus-secreting goblet cells (22) – aiding the entrapment and further removal of unwanted inhaled particles (23). Two types of cells make up the alveolar epithelium; type 1 cells, which facilitate gaseous exchange, and the type 2 cells produce numerous secretory vesicles filled with surfactant material, including surfactant-associated protein C (24). Thus, in a normal lung the architectural structure of the tissue works to provide the most efficient environment for gaseous exchange.

Due to the large surface area and constant barrage of pathogens and debris found in the air, the lungs have developed efficient mechanisms for the recognition of microbe-specific motifs. The respiratory tract is also unique in that it has both an external epithelial layer (the respiratory epithelium) and an internal endothelial layer in close apposition. Therefore, this unique structure could provide difficulties when attempting to pharmacologically target the tissue resident eosinophils rather than the airway-resident cells.

### Neutrophil-dominant airway inflammation

In tissue localized infection, the exposure of neutrophils to bacterial products or endogenous mediators leads to “priming” of function and facilitates chemotaxis toward the site of infection or injury. Up regulation of surface adhesion molecules (P-selectin, ICAM1, and VCAM1) on the vascular endothelial cells that interact with adhesion molecules on the neutrophil is required for the tethering, rolling, intravascular crawling, and transmigration of activated neutrophils from the circulation into the tissue to carry out their effector functions [reviewed in Ref. (4)]. Development and progression of two neutrophil-driven airway diseases; COPD, characterized by impaired airflow to the lungs as a result of an abnormal inflammatory response (25), and bronchiectasis, a chronic debilitating respiratory disease, characterized by a “vicious cycle” of permanently dilated airways, increased mucus production, and recurrent infections (26), have been linked to failed resolution of inflammation (27–29). However, despite persistent neutrophil-driven inflammation, reduced bacterial clearance is also seen (30). Thus, failure to clear bacterial pathogens from the airways leads to a prolonged inflammatory response characterizing the vicious cycle of inflammation and infection described, with both neutrophil and bacterial derived products contributing to damage of the surrounding epithelial cells.

Currently prescribed treatments for COPD and bronchiectasis include β2-adrenergic receptor agonists (e.g., salmeterol and formoterol), anticholinergic therapies (e.g., tiotropium bromide), high dose inhaled glucocorticoids, theophylline and treatments to improve mucociliary clearance, and sputum expectoration. These drugs work to reduce symptoms, improve lung function, and exercise capacity in an attempt to return to normal health status (26, 31–34). Furthermore, as well as traditional anti-inflammatory effects including inhibition of ROS release, decreased adhesion to the vascular endothelium and reduced release of pro-inflammatory cytokines from macrophages (35), salmeterol has also been shown to reduce adherence of bacteria to airway epithelial cells (36, 37), demonstrating that it may be effective at treating both the underlying infections and resultant inflammatory response.

### Eosinophil dominant airway inflammation

**Figure 1:**
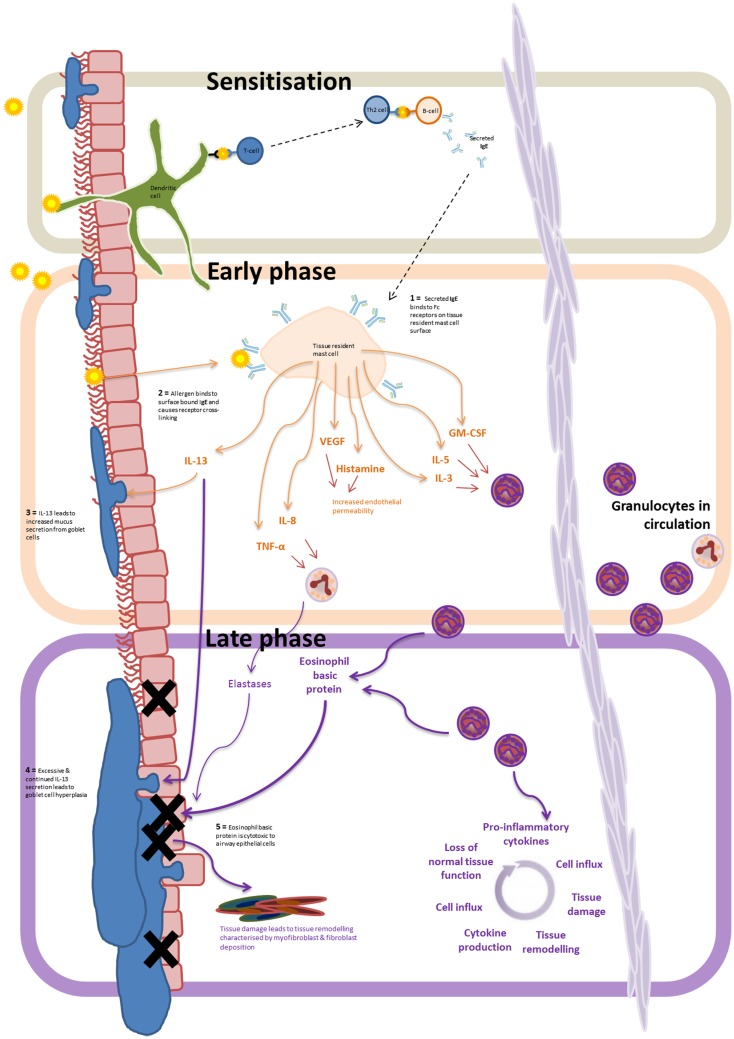
**Allergen driven allergic inflammation: progression from the sensitization phase, to early and late inflammatory phase**. Airway exposure results in the allergen being taken up by submucosal dendritic cells. Antigen presentation to B cells, via T cell–dendritic cell interactions, leads to IgE release – marking the sensitization phase. IgE binding to Fcε receptors on the tissue resident mast cells leads to allergen-induced IgE receptor crosslinking and mast cell degranulation – marking the early-phase of the inflammatory response. The release of of LTB_4_, TNF-α, IL-8, IL-13, CCL2, and VEGFA from mast cells results in eosinophil recruitment, increased vascular permeability, and increased mucus secretion by goblet cells. Continued exposure to the allergen, and infiltration of granulocytes to the inflamed tissue marks the progression to the late phase of the inflammatory response. Prolonged secretion of IL-13 and the release of intracellular cytotoxic granules by recruited eosinophils (and neutrophils) lead to continual tissue damage, mucus hypersecretion, and tissue remodeling resulting in the gradual loss of normal lung function.

Eosinophil dominant allergic inflammation is characterized by three distinct phases (Figure 1). On initial exposure of the airway to an allergen, the sensitization stage, allergens are taken up by dendritic cells either within the airway lumen or in the submucosa after penetrating the epithelial barrier. The antigens are then presented to naïve T cells, which differentiate and activate local B cells to produce IgE. Secreted IgE then binds to Fcε receptors on the surface of submucosal tissue resident mast cells, thus priming the immune system. On second exposure to the allergen, surface bound IgE becomes cross-linked leading to the activation of the tissue resident mast cells. Inflammatory mediators are then released and initiate the propagation of inflammation characterized as the second phase of allergic inflammation, the early-phase reaction. Release of histamine, LTB_4_, TNF-α, IL-8, IL-13, CCL2, and VEGFA from mast cells leads to increased vascular endothelial permeability, promoting the recruitment and transmigration of granulocytes from the circulation into the tissue. IL-13, histamine and TNF-α also act directly on the goblet cells found within the airway epithelium, causing increased mucus production.

Transendothelial migration of eosinophils to the inflamed site marks the progression into the third stage of the inflammatory response – the late-phase reaction. This usually develops 6–9 h after allergen exposure. Continued secretion of eosinophil recruiting cytokines (e.g., GM-CSF, IL-5, and IL-3) by mast cells leads to the prolonged eosinophil infiltration, representing a major contributory factor to the initiation and maintenance of eosinophilic airway inflammation in asthma (19, 38) – key cytokines involved in eosinophil recruitment are summarized in Table 1. Subsequent eosinophil degranulation and release of intracellular cytotoxic contents such as eosinophil basic protein results in damage to airway epithelial cells with increased mucus production from goblet cells and airway bronchoconstriction as a result of IL-13 secretion lead to reduced airflow, airway damage, goblet cell hyperplasia, and disrupted tissue architecture and remodeling. Mast cell production of IL-8 and TNFα also triggers the recruitment of neutrophils and elastase release causing further tissue degradation.

**Table 1:** **Key cytokines involved in eosinophil recruitment**.

**KEY CYTOKINES**
Eotaxin-1 (CLL11) (47, 48)
GM-CSF (49)
Interleukin-5 (IL-5) (49)
Interleukin-3 (IL-3) (38, 49)
MCP-3 (48)
Eotaxin-2 (CCL24) (47)
RANTES (CCL5) (48–50)
MIP-1α (CCL3) (48, 50)

Continuous exposure to allergens leads to persistent, chronic inflammation, which is associated with changes in tissue architecture and cell composition and extensive tissue remodeling. In particular, patients with chronic asthma develop increased goblet cell hyperplasia. Persistent residence of eosinophils and neutrophils within the submucosa results in the continuous production of pro-inflammatory cytokines, lack of inflammatory resolution, and a repetitive cycle of tissue injury and inflammation (Figure 1).

The most characteristic features of eosinophil dominant allergic asthma is bronchial inflammation leading to non-specific airway hyperreactivity (39), mucus plugging of airways, epithelial cells loss, mucus gland hyperplasia, epithelial basement membrane thickening, edema of the submucosa, smooth muscle hypertrophy, and inflammatory cell infiltration (40). *In vitro* studies have shown that eosinophil granule-derived proteins are partly responsible for the damage seen in asthma as eosinophil cationic protein and major basic protein are both cytotoxic to the bronchial epithelium (16) and have been shown to affect ciliary beat and function (41) and increase non-specific bronchial hyperreactivity (42), all of which are classical pathological findings of asthma (40). Interestingly, a number of studies using eosinophil-deficient mice (PHIL) have shown that eosinophils enhance airway mucus accumulation and hyperresponsiveness, collagen deposition, and smooth muscle hypertrophy (43, 44). However, a significantly increased mucus index was still observed in ovalbumin treated PHIL mice (43) suggesting that although eosinophils contribute substantially to airway remodeling, they are not obligatory for allergen-induced injury (44), indicating activation of both eosinophil-dependent and -independent mechanisms of injury after airway allergen challenge (43).

Current treatments for asthma include inhalable bronchodilators (β2-adrenergic agonists and anticholinergic drugs), leukotriene receptor antagonists, glucocorticoids, and theophylline (45, 46). The possible mechanisms of actions for these drugs are shown in Table 2.

**Table 2:** **Mechanisms of action of agents currently used for routine treatment of allergic airways disease**.

Mediator/drug	Biological response	Reference
Glucocorticoids	Alter pro- and anti-inflammatory cytokine balance	(55, 56)
	Enhances phagocytic capacity of macrophages and airway epithelial cells	(57–59)
	Promotes eosinophil apoptosis *in vitro* possibly via reduction of Mcl-1 levels	(1, 60)
β2-Adrenergic receptor agonists (e.g., salmeterol and formoterol)	Highly selective bronchodilator Reduce adherence of bacteria to airway epithelial cells	(61–63) (36, 37)
Anticholinergic drugs (e.g., tiotropium bromide)	Effective bronchodilators	(32)
Leukotriene receptor antagonists	Prevent leukotriene induced bronchoconstriction, mucus hyper secretion, and airway inflammation	(64)
	Increase eosinophil apoptosis *in vitro*	(65)
Theophylline	Bronchodilator	(66, 67)
	Accelerates eosinophil apoptosis *in vitro* possibly by suppressing anti-apoptotic protein Bcl-2 levels	(68, 69)
	Reduced airway eosinophilia and ECP levels *in vivo*	(70)

Mast cells are known to play an important role in the propagation and pathogenesis of allergen-induced inflammatory disease (51, 52), due to their involvement in the sensitization stage, Figure 1. As these tissue resident cells are long lived and can survive repeated activation, the modulation of their proliferation and survival has been proposed as a potential therapeutic intervention for allergic disease (53). Recently, Wechsler et al. showed that thymol, a monocyclic phenolic plant compound with known antiseptic, antibiotic, antifungal, and antioxidant properties, was able to induce mast cell apoptosis *in vitro* and *in vivo* (54), thereby highlighting a potential pathway for modulating the allergic response through the manipulation of mast cell viability.

## Failings in Current Treatments: Why New Approaches are Needed?

New approaches for the management of inflammatory airway diseases are urgently needed as current treatments are associated with a number of adverse health consequences after long-term use. For example, the management of allergic asthma is largely based around preventing exposure of the sensitized individuals to the allergen and treating with therapies which are directed toward alleviating and/or treating the symptoms of the disorder, such as inhaled glucocorticoids. However, this often poses a problem as a small subpopulation of asthma sufferers, often those with “neutrophilic” asthma, are noted to be steroid resistant (71), resulting in increased disease severity (72). Equally the undesirable side effects that arise from long-term use of glucocorticoids (the most common treatment prescribed for eosinophil dominant inflammatory conditions) include osteoporosis, hypertension, muscle atrophy, and delayed wound healing, all of which place limitations on use of glucocorticoid-based anti-inflammatory therapies.

### Regulation of granulocyte apoptosis

As the resolution of inflammation likely depends on the apoptosis and phagocytosis of apoptotic granulocytes, research into the pharmacological manipulation of these processes is increasingly being recognized as an important area of research for the development of novel strategies to enhance the resolution of chronic inflammation (27, 73–76) and improve patient health.

The rates of granulocyte apoptosis are amenable to alteration by exogenous pharmacological compounds. Both the rates of neutrophil and eosinophil apoptosis can be accelerated by treatment with, soluble Fas ligand (Fas-L) (77), gliotoxin (78), and cyclin-dependent kinase inhibitors (CDKi) (73, 74, 79). Neutrophil apoptosis can also be delayed by pro-inflammatory cytokines (e.g., TNF-α and IL-1) (78), bacterial products [e.g., lipopolysaccharide (LPS), lipoteichoic acid, and peptidoglycan] (80, 81), growth factors [e.g., granulocyte macrophage-colony stimulating factor (GM-CSF)] (75), and pharmacological agents including dibutyryl-cAMP (82, 83) and glucocorticoids (1, 84). TNF-α has been reported to have both pro- and anti-apoptotic effects on neutrophils, with early apoptosis and late survival seen during exposure of cultured cells. It is thought that at early time points (2–8 h) during *in vitro* culture a subpopulation of neutrophils undergo caspase-8 dependent apoptosis, with later survival (16–24 h) dependent upon an NF-κB mediated anti-apoptotic signaling pathway. Similarly, NF-κB inhibition in eosinophils allows TNF-α mediated apoptosis to predominate (85).

One important difference between the two granulocyte populations is that *in vitro* treatment of human granulocytes with glucocorticoids promotes eosinophil apoptosis, whereas it delays neutrophil apoptosis (5). It is also important to note that the survival effect of glucocorticoids on neutrophil longevity may be dependent on the environmental milieu (86, 87). Marwick et al. demonstrated *in vitro* that the pro-survival effects of glucocorticoids on neutrophils are dependent on oxygen levels, with severe hypoxia (1% oxygen) attenuating glucocorticoid-mediated neutrophil survival (87). This observation has important implications for the therapeutic efficacy of glucocorticoids when prescribed for neutrophil-dominant inflammatory conditions, due to the relatively high oxygen concentrations found in the lung.

One way that glucocorticoids are thought to mediate their anti-inflammatory effects is through the expression and function of the downstream effector molecule Annexin A1 (AnxA1) (86, 88). AnxA1 has been shown to promote human neutrophil apoptosis via dephosphorylation of the Bcl-2-antagonist of cell death (BAD) promoting cell death via the intrinsic pathway of apoptosis (89). *In vitro* investigations showed that endogenous AnxA1 was released by apoptotic neutrophils and glucocorticoid-treated macrophages, which then acts in both a para- and autocrine manner to promote the phagocytic clearance of apoptotic neutrophils (90, 91). Increased production of AnxA1 by innate immune cells following glucocorticoid administration reportedly leads to decreased neutrophil endothelial transmigration, increased neutrophil apoptosis and increased phagocytosis of apoptotic cells by macrophages (88, 92). This mechanism was further supported by *in vivo* experiments in which administration of an anti-AnxA1 antibody prevented glucocorticoid-induced resolution of inflammation, whereas treatment with AnxA1-derived peptides promoted the resolution of inflammation (86). Further work is required to define the role of AnxA1 in the resolution of eosinophilic inflammation.

A number of classically “pro-inflammatory” eosinophil recruiting cytokines, IL-25 (93), IL-33 (94), IL-3, IL-5, and thymic stromal lymphopoietin (TSLP) (95) have also been shown to delay the rate of eosinophil apoptosis (96). Due to their key role in the recruitment and activation and of eosinophils in inflammatory sites, there is interest in developing anti-cytokine therapies, such as anti-IL-5 antibodies [reviewed in Ref. (97)], as potential therapeutic targets. Historically, there has been a number of disappointing results surrounding anti-IL-5 treatments in humans, potentially as a result of the unique architecture of the lungs localizing their effect to the airway resident, rather than the tissue resident, eosinophils.

### Novel regulators of eosinophil apoptosis

The regulation of cell apoptosis by pro- and anti-apoptotic Bcl-2 family members has been well documented (20). However, as granulocytes have a limited number of mitochondria (98), it was somewhat surprising when members of this protein family were found to modulate the regulation of granulocyte apoptosis. Eosinophils were found to express high levels of pro-apoptotic Bax molecules, and were also found to express a number of anti-apoptotic members of the Bcl-2 family (99). Mcl-1, an important anti-apoptotic protein in neutrophils (27), is also thought to play a predominant role in eosinophils, as previous work reported that Mcl-1 levels decreased in glucocorticoid-treated eosinophils, whereas they remained at a constant level in glucocorticoid-treated neutrophils (60), which may go some way to explain their differential effect on the two granulocyte populations.

Since granulocytes are considered to be terminally differentiated cells, the central role of active cyclin-dependent kinases (key regulators of the cell cycle) in the control of apoptosis was surprising. The structurally distinct CDKis R-roscovitine and AT7519 promoted apoptosis in a caspase-dependent manner (73, 76) by the down-regulation of intracellular Mcl-1 levels *in vivo* (79, 100, 101) and prevented GM-CSF-mediated up regulation of Mcl-1 (73). R-roscovitine was also shown to have pro-resolving effects *in vivo* in a number of models of inflammation (73) while AT7519 increased the percentage of apoptotic eosinophils as well as the percentage of macrophages containing apoptotic eosinophils in a mouse model of allergic pleurisy, indicating that AT7519 has the potential to resolve allergic inflammation by driving both eosinophil apoptosis and by increasing macrophage clearance of apoptotic cells (76). Evidence suggests that these CDKIs target CDK7 and CDK9, which are involved in transcription of key granulocyte survival proteins such as Mcl-1 (102). Further studies investigating the mechanisms underlying resolution of inflammation have also highlighted the importance of Mcl-1 in the regulation of granulocyte apoptosis. Flavones, polyphenolic plant-derived compounds, rapidly induced both eosinophil (103) and neutrophil apoptosis (101) *in vitro* even in the presence of powerful pro-survival mediators including LTA, GM-CSF (101), and IL-5 (103).

Another powerful driver of caspase induced eosinophil apoptosis is antibody crosslinking of sialic acid binding immunoglobulin-like lectin 8 (Siglec-8), a member of the Siglec immunoglobulin supergene family expressed only on the surface of human eosinophils, basophils, and mast cells (104). Siglec-8 cross-linking reduced eosinophil viability in a time- and concentration-dependent manner through the induction of caspase-mediated apoptosis. This was further confirmed by the use of pan (104) and selective caspase inhibitors (against caspase-8 and -9) (105), which completely inhibited Siglec-8 cross-linking induced apoptosis *in vitro*, while having no effect on spontaneous eosinophil apoptosis. Antibody crosslinking of the functional mouse ortholog, Siglec-F, was also shown to significantly reduce peripheral eosinophil number in a hypereosinophilic/chronic eosinophilic leukemic (HES/CEL) murine model, as well as induce eosinophil apoptosis *in vivo* (106). These data further highlight that regulation of eosinophil apoptosis using exogenous mediators could provide potential future therapeutic targets for eosinophilic disorders.

Pharmacological modulation of endogenous molecules involved in mediating the resolution of allergic inflammation is also a key area of research. Recently, Faustino et al. showed that tumor necrosis factor-related apoptosis-inducing ligand (TRAIL) is a major player in the resolution process, with *in vivo* administration of anti-TRAIL markedly reducing the number of apoptotic cells in the BAL fluid of chronic allergy induced mice (107). *In vivo*, treatment with recombinant TRAIL, in an established mouse model of allergic airway inflammation, also significantly augmented the number of apoptotic cells found in the BAL of OVA treated mice, compared to control (PBS treated mice), as well as decreasing the overall number of eosinophils found in the BAL (107), providing further evidence that the manipulation of eosinophil apoptosis may provide avenues for the discovery of novel therapeutics.

### Lipid modulation

Lipoxins, protectins, and resolvins are bioactive lipids synthesized from arachidonic, docosahexaenoic, and eicosapentaenoic acid, respectively (108, 109). They are key pro-resolution mediators, which act to selectively prevent granulocyte migration and increase the recruitment of phagocytic cells (110). Both lipoxin A4 and B4 were reported to inhibit neutrophil recruitment to an inflammatory site and lipoxin A4 was also shown to stimulate monocyte chemotaxis and promote macrophage uptake of apoptotic neutrophils *in vitro* (111, 112) and *in vivo* (113). Similar inhibitory effects of lipoxin A4 upon the migration and chemotaxis of eosinophils *in vivo* and the local generation of eotaxin and IL-5 have been reported (114, 115). Resolvins also have pro-resolution effects preventing transepithelial and transendothelial migration of neutrophils *in vivo* (116), and stimulating the non-inflammatory phagocytosis of apoptotic neutrophils (117).

The significance of pro-resolving lipids in successful resolution of inflammation has also been noted in a number of non-allergic and allergic inflammatory conditions. Reduced levels of protectin D1 and lipoxin A4 are seen in the exhaled breath of patients after a severe and mild asthma exacerbation, respectively (118, 119). *In vivo*, mouse models have also provided insight into the role of pro-resolving lipids in the recruitment of granulocytes and augmentation of macrophage phagocytic capacity – resolvin E1 and lipoxin analogs were shown to reduce airway hyperresponsiveness (119), eosinophil number (118), and promote inflammation resolution in a mouse model of allergic asthma (119, 120). Given the pro-resolution roles of these lipid mediators, there is great interest in the development of them as therapeutics. However, as these endogenously produced molecules are traditionally locally active and rapidly inactivated the development of exogenously administered drugs with longer half-lives, which mimic endogenous compounds *in vivo* are needed to fill this pharmacological niche. Currently, the synthetic resolvin analog RX-10045, and naturally occurring small molecule lipid mediator RX-10001 and are under clinical examination for their use in a number of inflammatory diseases such as dry eye, asthma, retinal disease, and inflammatory bowel disease (109).

### Manipulation of phagocytosis

One newly emerging approach to facilitate the resolution of inflammation is the pharmacological manipulation of the phagocytosis of apoptotic granulocytes (121). As well as their effects on granulocyte apoptosis, glucocorticoids are also known to augment macrophage phagocytic function (57), which may represent an approach to drive clearance of apoptotic cells from inflamed sites (122). Glucocorticoid-treated macrophages exhibit altered cytoskeletal regulation, with increased cell motility and expression of high levels of active Rac, a key protein involved in cell motility, mitosis, wound healing, and phagocytosis (123).

Glucocorticoids induce phagocytosis of apoptotic cells via Mer, a member of the Tyro-3/Axl/Mer (TAM) receptor tyrosine kinase family (58, 124, 125). TAMs are widely expressed vertebrate-specific receptor tyrosine kinases that confer the capacity for binding and subsequent phagocytosis of apoptotic cells, together with initiation of signals that regulate cellular function. TAM-deficient mice show defective clearance of apoptotic material by retinal pigment epithelial cells of the eye, Sertoli cells of the testis and also by myeloid cells. Interestingly, a number of autoimmune conditions are associated with impaired or failed clearance of apoptotic cells (126) and the absence of TAM receptors results in progressive loss of vision, reduced fertility, and development of overt autoimmunity (122, 127, 128). Recent studies demonstrate that Mer-mediated apoptotic cell clearance has a critical importance pathophysiologically in the lung, as inflammation in an LPS-induced lung injury model was amplified following Mer blockade (129), and conversely attenuated following up regulation of Mer-signaling by use of TAPI-0 (a specific inhibitor of Mer cleavage) (130). The expression of Mer on phagocytic populations present at the inflammatory site could also be induced by treatment with glucocorticoids (57) or liver X receptor agonists (131). Alternatively, blockade of cytokines that actively suppress Mer expression (e.g., interferon-gamma) could represent an alternative strategy for promoting Mer-dependent apoptotic cell clearance (132, 133). Recent evidence suggests that Mer is down regulated by inflammatory stimuli such as LPS or bleomycin via proteolytic cleavage from the phagocyte membrane (134). Specific inhibition of ADAM17 proteolytic activity (e.g., using KD-1X-73.5 or TAPI-0) prevents Mer down-regulation and is associated with increased clearance of apoptotic cells in both LPS and bleomycin models of lung injury (135), providing a potential therapeutic approach to increase Mer-dependent clearance mechanisms in inflammation. Definition of the molecular mechanisms of phagocyte–apoptotic cell interactions and regulation by glucocorticoids will provide opportunities to identify novel targets for therapeutic gain.

### Epithelial cell phagocytosis

In addition to the importance of therapies, which are able to modulate apoptotic cell phagocytic clearance, identification of the cell types which carry out this process in inflammatory airway conditions is of crucial importance. Induced death of airway epithelial cells as a result of exposure to environmental toxins, allergens, and pathogens has been observed and documented in patients with asthma (3). Thus, there is a need for a large population of local airway phagocytic cells to remove the apoptotic debris. There is mounting evidence that a number of “non-professional” phagocytes, including mammary epithelial (136) and microvascular endothelial cells (137) are also able to phagocytose apoptotic cells. Work published by Walsh et al. and Sexton et al. showed that bronchial epithelial cells are capable of recognizing and engulfing apoptotic eosinophils, suggesting a non-passive role of the airway epithelium in the resolution of eosinophilic inflammation in asthma (138, 139). More recently, Juncadella et al. showed that bronchial epithelial cells are also critically involved in the phagocytosis of apoptotic airway epithelial cells, which subsequently alters the production of anti-inflammatory cytokines and control of airway hyperresponsiveness in a murine model of allergic airway inflammation (3). Despite the potential for providing novel therapeutic approaches for the treatment of inflammatory diseases, little work has been done to investigate the potential for manipulation of the phagocytic ability of these cells in current models of inflammatory airway diseases.

## Summary

In conclusion, recent research into the pharmacological manipulation of apoptosis and efferocytosis of apoptotic cells has provided novel insights into the treatment of inflammatory airway diseases, notably eosinophil dominant airway inflammation. This dual approach will open up new areas for therapeutic intervention, allowing the successful manipulation of inflammation resolution, as well as reducing the adverse effects associated with currently available treatments.

## Conflict of Interest Statement

The authors declare that the research was conducted in the absence of any commercial or financial relationships that could be construed as a potential conflict of interest.
